# Supplementary material for: Vesicular glutamate release from central axons contributes to myelin damage
Source: Nat Commun. 2018 Mar 12;9:1032. doi: 10.1038/s41467-018-03427-1 (PMC5847599; doi:10.1038/s41467-018-03427-1)
Supplement: Supplementary file 1 — Supplementary information [file 41467_2018_3427_MOESM1_ESM.pdf]

Supplementary material

Doyle et al

NCOMMS-1-02047D

8 Supplementary Figures

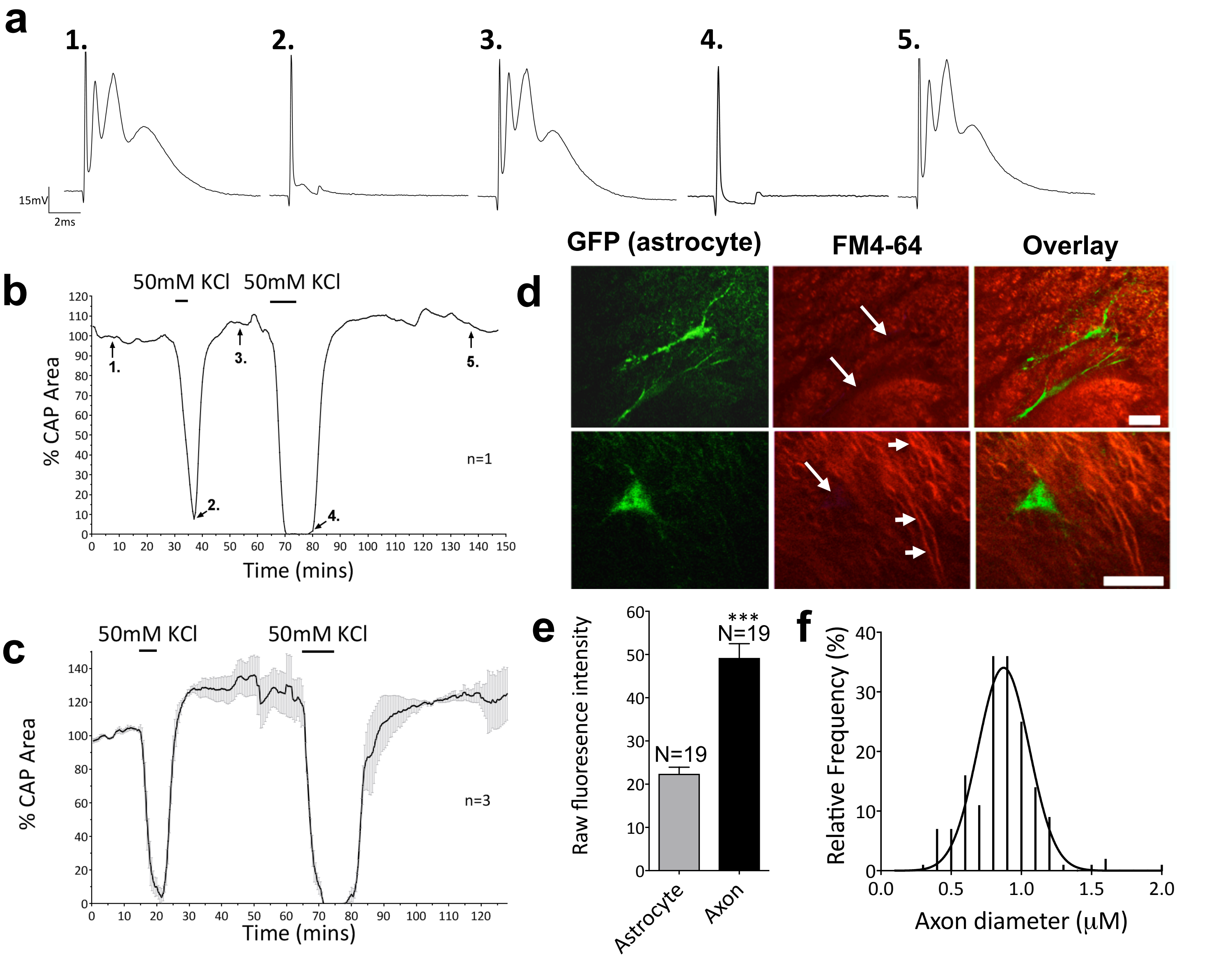

Supplementary Figure 1. Effects of [K+]-evoked depolarization on WM axons and glia. a-c: CAP recordings from adult rat RON (a, b) and CC (c) showing CAP failure during perfusion with elevated [K+]. d: Examples of astrocytes (green) in FM4-64 loaded GFAP-GFP CC. Note the low level of astrocyte FM4-64 (red) staining (arrows), while surrounding axons express high levels of FM fluorescence (arrow heads). Scale = 10 µm. e: Mean FM4-64 pixel intensity in astrocytes is significantly lower than in axons. Unpaired t-test P = 0.0001. f: YFP expressing axon diameter spectrum for 167 axons measured at random from 45 brain slices.

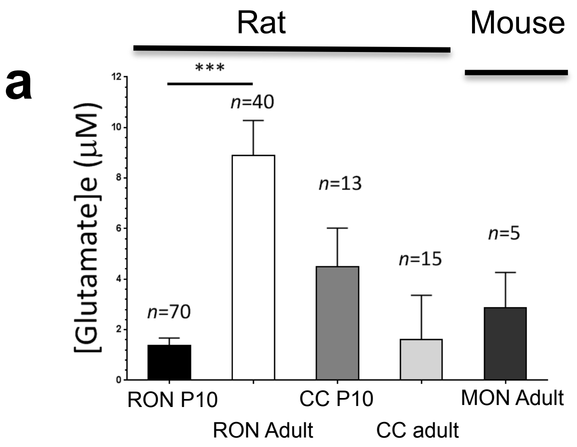

Supplementary Figure 2. Resting $[glutamate]_e$ differs between WM structures and tonic glutamate uptake maintains low $[glutamate]_e$. ANOVA with Holm- Šídák, *** = 0.0001. a: Resting $[glutamate]_e$ in various WM preparations. b: The effects of glutamate transport block with 200 μM TBOA on mean $[glutamate]_e$ in adult CC. Unpaired t-test, * = 0.046. c: The effects of glutamate transport block on P10 rat RON $[glutamate]_e$. Unpaired t-test, *** = 0.0001.

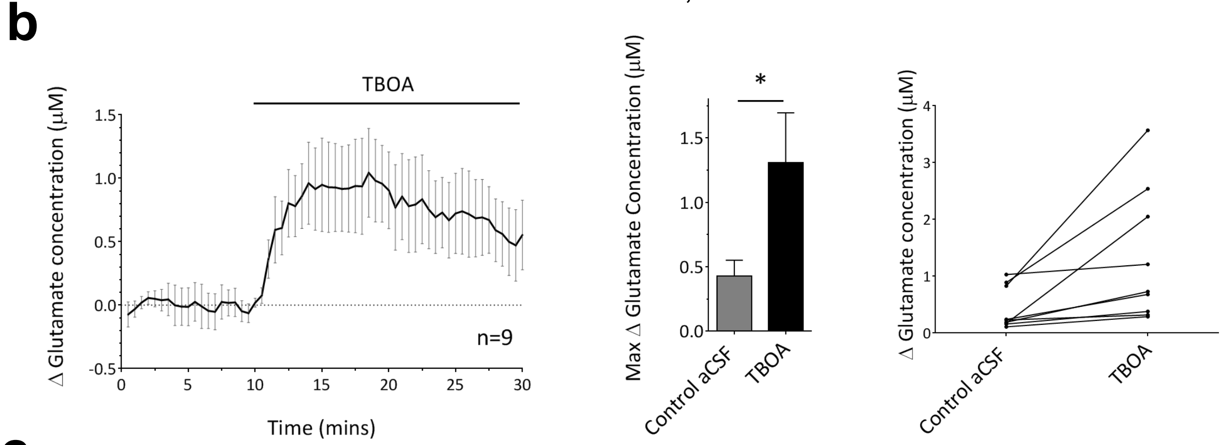

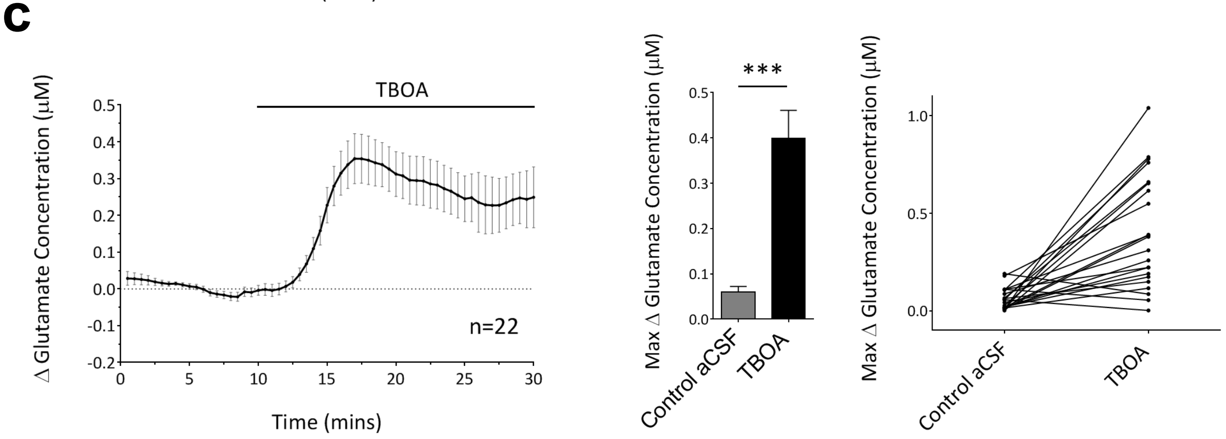

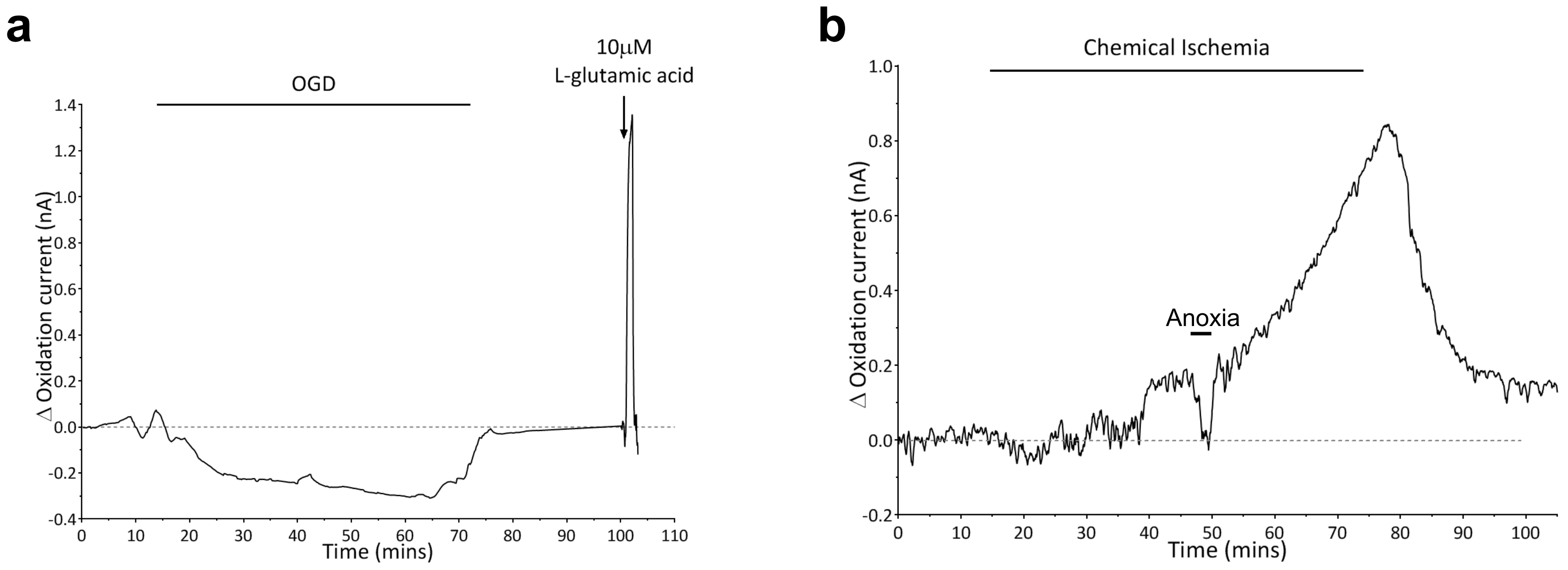

Supplementary Figure 3. Glutamate biosensors are anoxia-sensitive. a: Differential electrode current ([glutamate]$_e$) measured in adult CC shows a paradoxical decline during OGD. b: During ischemic conditions (antimycin-a/0-glucose) a brief period of anoxia reverses ischemia-induced current.

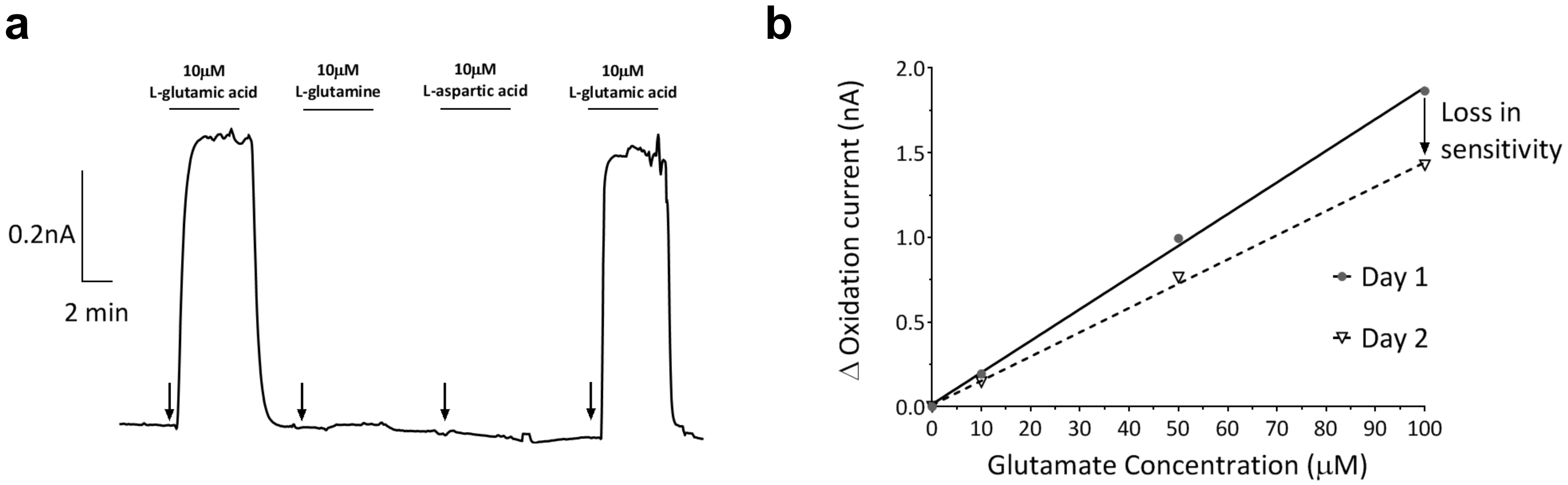

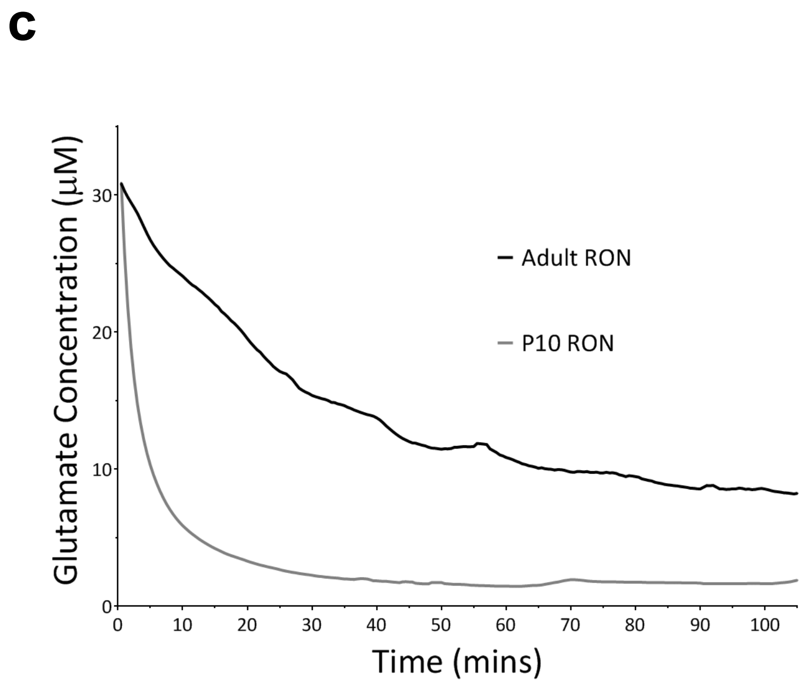

Supplementary Figure 4. Glutamate biosensor recordings. a: Selectivity and reproducibility of glutamate electrode recording. b: Calibration on subsequent days showing gradual loss of sensitivity but retention of linear calibration. c: Example of elevated $[\text{glutamate}]_e$ in adult and neonatal rat RON (recordings initiated 20 min after electrode insertion).

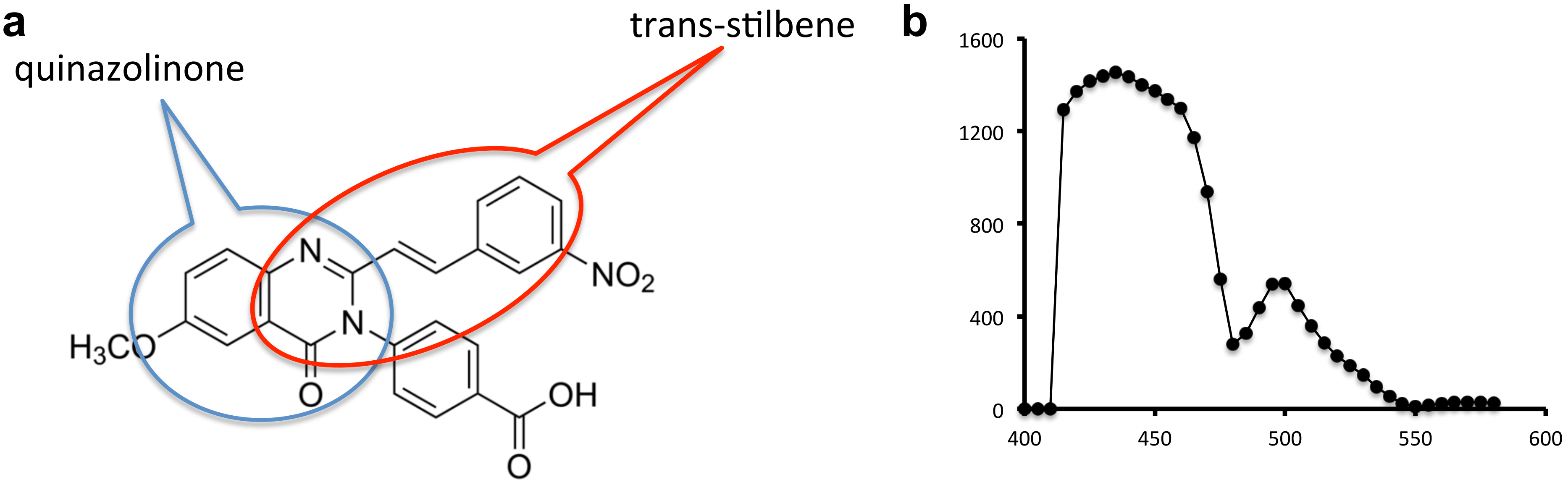

c

Lipinski's rule of five *	conforms
Topological polar surface area *	122.34 A^2
cLogP *	4.45
LogBB **	-0.995

* ChemAxon, Budapest, Hungary
** Clark, 1999.

Supplementary Figure 5. QNZ-46 characteristics. a: Molecular structure, indicating fluorescent (blue) and myelin-targeting (red) regions. b: Lambda scan of the fluorescent emission following excitation at 405 nm (10 mM QNZ-46 in immersion oil). c: Biophysical properties.

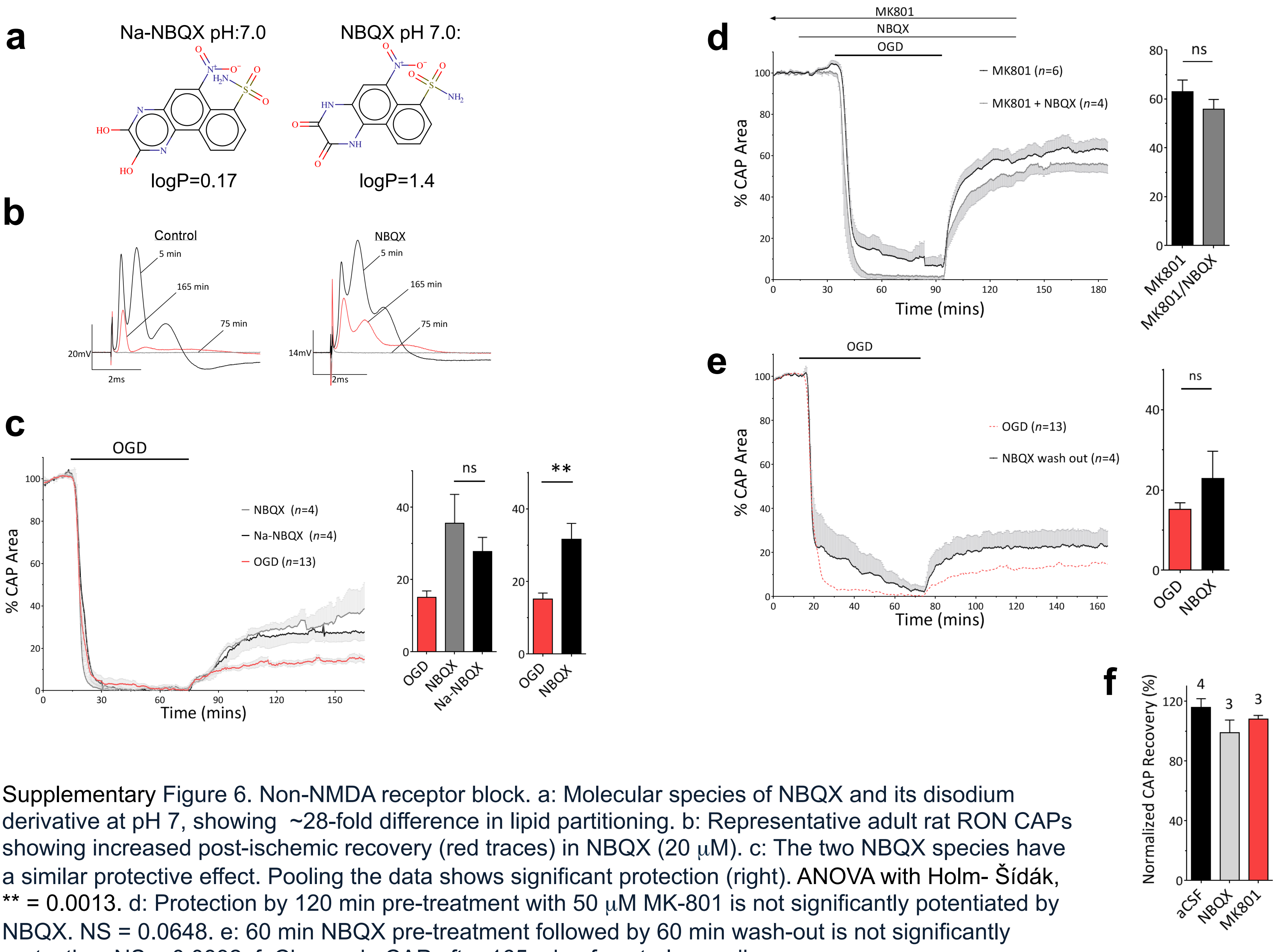

Supplementary Figure 6. Non-NMDA receptor block. a: Molecular species of NBQX and its disodium derivative at pH 7, showing ~28-fold difference in lipid partitioning. b: Representative adult rat RON CAPs showing increased post-ischemic recovery (red traces) in NBQX (20 μM). c: The two NBQX species have a similar protective effect. Pooling the data shows significant protection (right). ANOVA with Holm- Šídák, ** = 0.0013. d: Protection by 120 min pre-treatment with 50 μM MK-801 is not significantly potentiated by NBQX. NS = 0.0648. e: 60 min NBQX pre-treatment followed by 60 min wash-out is not significantly protective. NS = 0.0832. f: Change in CAP after 165 min of control recording.

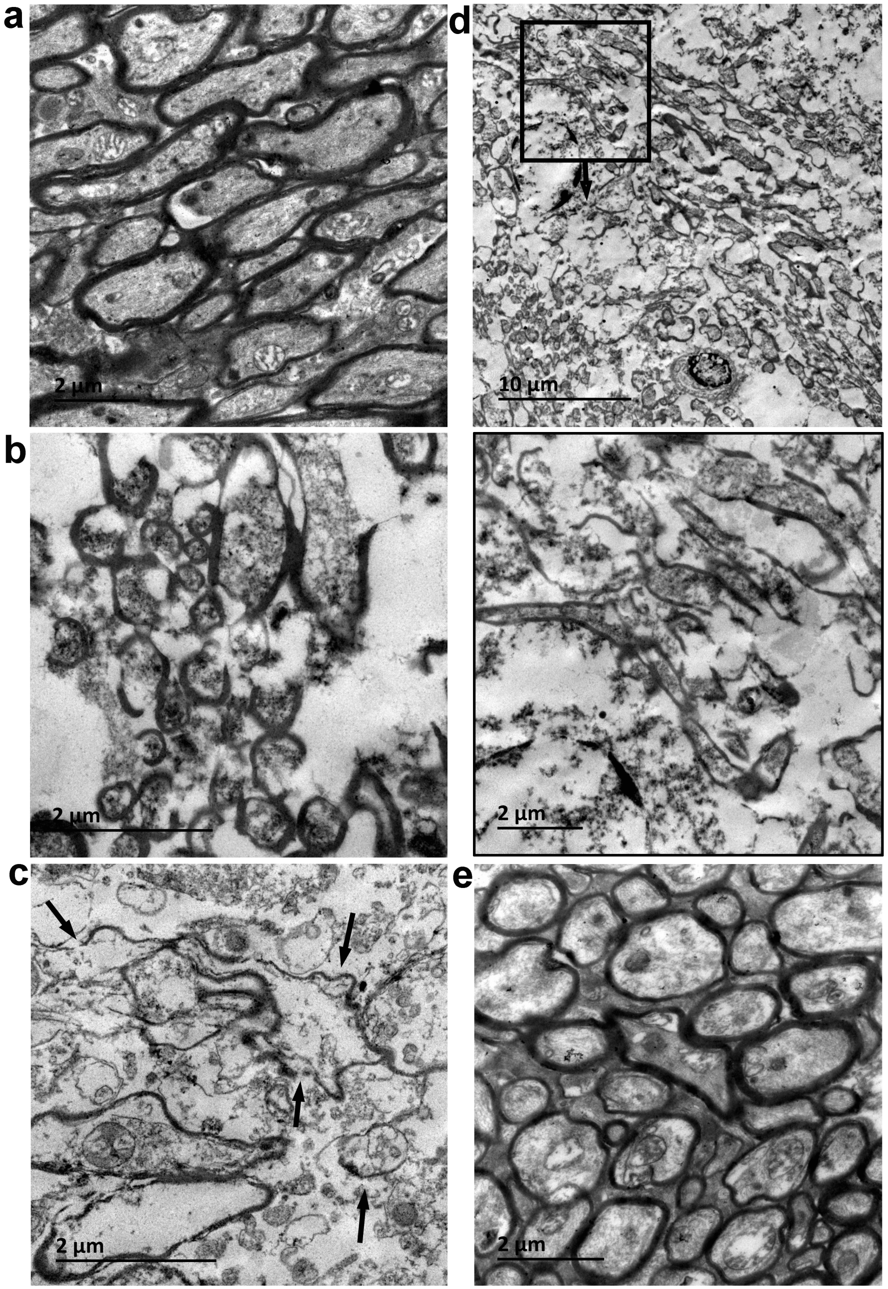

Supplementary Figure 7. Ultrastructural evidence for myelin damage. a: Myelinated axons from contralateral external capsule-motor cortex border. b: Similar region from the ipsilateral side showing discontinuous and disrupted myelin profiles. c: Areas of myelin separation and decompaction (e.g. arrows) were found throughout the ipsilateral side. d: Lower power image showing wide-scale myelin disruption with the boxed area at higher gain below. e: Normal appearing myelin profiles in the same ipsilateral region following treatment with QNZ-46.

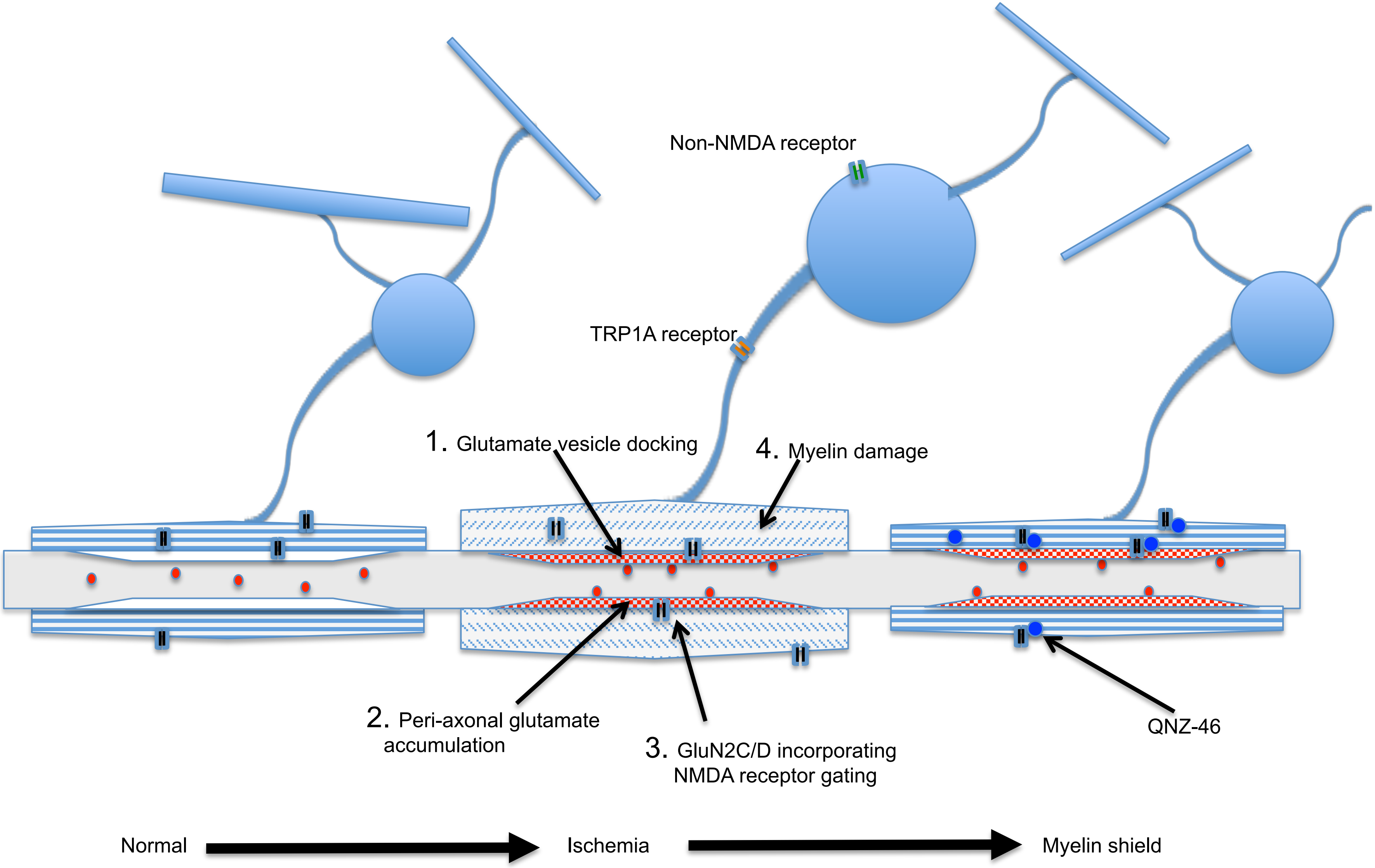

Supplementary Figure 8. Diagram showing the mechanisms of myelin damage and action of myelin shield. In physiological conditions (left) compact myelin contains GluN2C/D-incorporating NMDA receptors at high density and ensheaths axons containing glutamatergic vesicles (red). There is a peri-axonal space under the myelin with limited access to the extracellular space. During ischemia (center), axonal glutamate vesicles dock with the axolemma releasing glutamate largely into the peri-axonal space and activating myelinic NMDA receptors. Myelin rapidly decompacts and function is lost. QNZ-46 penetrates myelin and is retained, preventing myelinic NMDA receptor activation with high selectivity and preventing myelin damage.
